# Cyclo­hexane-1,2-diammonium bis­(pyridine-2-carboxyl­ate)

**DOI:** 10.1107/S1600536809038689

**Published:** 2009-09-30

**Authors:** Nam-Ho Kim, In-Chul Hwang, Kwang Ha

**Affiliations:** a School of Applied Chemical Engineering, The Research Institute of Catalysis, Chonnam National University, Gwangju 500-757, Republic of Korea; b Institute of Basic Sciences, Pohang University of Science and Technology, Pohang 790-784, Republic of Korea

## Abstract

In the dication of the title salt, C_6_H_16_N_2_^2+^·2C_6_H_4_NO_2_^−^, the two ammonium groups are in the equatorial positions of the chair-shaped cyclo­hexyl ring. In the crystal, the cations and anions are linked by N—H⋯O and N—H⋯N hydrogen bonds, forming a layer network parallel to the *ac* plane. Weak π–π inter­actions between adjacent pyridine rings with a centroid–centroid distance of 3.589 (2) Å are also present.

## Related literature

For the syntheses and structures of cyclo­hexane-1,2-diammonium compounds, see: Lin & Lii (1998 ▶); Lin & Wang (2000 ▶). For the crystal structures of pyridine-2-carboxyl­ates, see: Kim & Ha (2009*a* ▶,*b* ▶,*c* ▶).
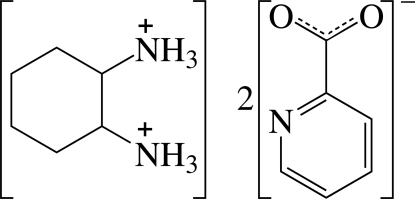


## Experimental

### 

#### Crystal data

C_6_H_16_N_2_^2+^·2C_6_H_4_NO_2_^−^
*M* *_r_* = 360.41
Monoclinic, 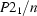

*a* = 9.2942 (11) Å
*b* = 20.329 (2) Å
*c* = 10.2189 (11) Å
β = 101.775 (3)°
*V* = 1890.1 (4) Å^3^
*Z* = 4
Mo *K*α radiation
μ = 0.09 mm^−1^
*T* = 293 K
0.20 × 0.10 × 0.10 mm

#### Data collection

Bruker SMART 1000 CCD diffractometer
Absorption correction: none
11005 measured reflections
3854 independent reflections
1741 reflections with *I* > 2σ(*I*)
*R*_int_ = 0.064

#### Refinement

*R*[*F* ^2^ > 2σ(*F* ^2^)] = 0.064
*wR*(*F* ^2^) = 0.171
*S* = 0.98
3854 reflections
237 parameters
H-atom parameters constrained
Δρ_max_ = 0.18 e Å^−3^
Δρ_min_ = −0.17 e Å^−3^

### 

Data collection: *SMART* (Bruker, 2000 ▶); cell refinement: *SAINT* (Bruker, 2000 ▶); data reduction: *SAINT*; program(s) used to solve structure: *SHELXS97* (Sheldrick, 2008 ▶); program(s) used to refine structure: *SHELXL97* (Sheldrick, 2008 ▶); molecular graphics: *ORTEP-3* (Farrugia, 1997 ▶) and *PLATON* (Spek, 2009 ▶); software used to prepare material for publication: *SHELXL97*.

## Supplementary Material

Crystal structure: contains datablocks global, I. DOI: 10.1107/S1600536809038689/ng2649sup1.cif

Structure factors: contains datablocks I. DOI: 10.1107/S1600536809038689/ng2649Isup2.hkl

Additional supplementary materials:  crystallographic information; 3D view; checkCIF report

## Figures and Tables

**Table 1:** Hydrogen-bond geometry (Å, °)

*D*—H⋯*A*	*D*—H	H⋯*A*	*D*⋯*A*	*D*—H⋯*A*
N1—H1*A*⋯O3	0.86	1.89	2.749 (3)	175
N1—H1*B*⋯O2	0.86	1.92	2.743 (3)	160
N1—H1*C*⋯O3^i^	0.86	2.10	2.790 (3)	137
N1—H1*C*⋯N4^i^	0.86	2.49	3.271 (4)	152
N2—H2*A*⋯O1^ii^	0.86	2.09	2.828 (3)	144
N2—H2*A*⋯N3^ii^	0.86	2.53	3.254 (4)	142
N2—H2*B*⋯O1	0.86	2.02	2.807 (3)	152
N2—H2*C*⋯O4^iii^	0.86	1.88	2.734 (3)	171

Symmetry codes: (i) 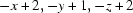; (ii) 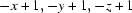; (iii) 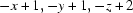.

**Fig. 1.**
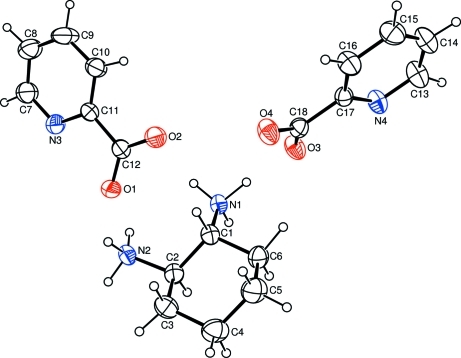
The structure of the title compound, with displacement ellipsoids drawn at the 30% probability level for non-H atoms.

**Fig. 2.**
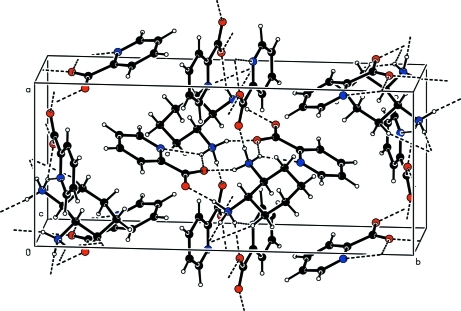
View of the unit-cell contents of the title compound. Hydrogen-bond interactions are drawn with dashed lines.

## supplementary crystallographic information

### Comment

The title compound, C_6_H_16_N_2_^2+^.2C_6_H_4_NO_2_^-^, consists of a doubly protonated cyclohexane-1,2-diammonium dication and two pyridine-2-carboxylate anions (Fig. 1). The dication has two chiral carbon atoms (C1 and C2), and is one of four possible stereoisomers. Both chiral atoms have *R* configuration. The cyclohexane ring of the dication adopts a strain-free chair conformation. The C—C—C bond angles lie in the range of 109.3 (3)°–111.6 (3)°, close to the ideal tetrahedral angle, and all neighboring C—H bonds are staggered. The diammonium groups within the dication are on opposite faces of the cyclohexane ring, that is, *trans* with respect to each other, and therefore the dication exists in the diequatorial conformation. The N1—C1—C2—N2 torsion angle of -59.0 (3)° displays the *gauche* conformation for the four atoms and there is a *gauche* interaction between the two NH_3_^+^ groups. The carboxylate groups of the anions appear to be delocalized on the basis of the C—O bond lengths [C—O: 1.239 (3)–1.255 (3) Å]. In the crystal structure, the component ions interact by means of many intermolecular N—H···O and N—H···N hydrogen bonds to form a two-dimensional network parallel to the *ac* plane (Table 1 and Fig. 2). There may also be intermolecular π-π interactions between adjacent pyridine rings, with a centroid-centroid distance of 3.589 (2) Å.

### Experimental

A solution of a mixture of *cis* and *trans* isomers of 1,2-diaminocyclohexane (0.202 g, 1.769 mmol) and pyridine-2-carboxylic acid (0.294 g, 2.388 mmol) in H_2_O (10 ml) was stirred for 3 h at 60 °C. The solvent was removed under vacuum and the residue was washed with ether/acetone/CHCl_3_, to give a white powder (0.112 g). Crystals suitable for X-ray analysis were obtained by slow evaporation from a CH_3_CN solution.

### Refinement

H atoms were positioned geometrically and allowed to ride on their respective parent atoms [C—H = 0.98 (CH), 0.97 (CH_2_) or 0.93 (aromatic) Å and N—H = 0.86 Å and *U*_iso_(H) = 1.2*U*_eq_(C, N)].

### Crystal data

**Table:**

C_6_H_16_N_2_^2+^·2C_6_H_4_NO_2_^−^	*F*(000) = 768
*M**_r_* = 360.41	*D*_x_ = 1.267 Mg m^−^^3^
Monoclinic, *P*2_1_/*n*	Mo *K*α radiation, λ = 0.71073 Å
Hall symbol: -P 2yn	Cell parameters from 784 reflections
*a* = 9.2942 (11) Å	θ = 2.3–17.2°
*b* = 20.329 (2) Å	µ = 0.09 mm^−^^1^
*c* = 10.2189 (11) Å	*T* = 293 K
β = 101.775 (3)°	Rod, colorless
*V* = 1890.1 (4) Å^3^	0.20 × 0.10 × 0.10 mm
*Z* = 4	

### Data collection

**Table:**

Bruker SMART 1000 CCD diffractometer	1741 reflections with *I* > 2σ(*I*)
Radiation source: fine-focus sealed tube	*R*_int_ = 0.064
graphite	θ_max_ = 26.4°, θ_min_ = 2.3°
φ and ω scans	*h* = −11→11
11005 measured reflections	*k* = −25→25
3854 independent reflections	*l* = −12→7

### Refinement

**Table:**

Refinement on *F*^2^	Primary atom site location: structure-invariant direct methods
Least-squares matrix: full	Secondary atom site location: difference Fourier map
*R*[*F*^2^ > 2σ(*F*^2^)] = 0.064	Hydrogen site location: inferred from neighbouring sites
*wR*(*F*^2^) = 0.171	H-atom parameters constrained
*S* = 0.98	*w* = 1/[σ^2^(*F*_o_^2^) + (0.0581*P*)^2^] where *P* = (*F*_o_^2^ + 2*F*_c_^2^)/3
3854 reflections	(Δ/σ)_max_ < 0.001
237 parameters	Δρ_max_ = 0.18 e Å^−^^3^
0 restraints	Δρ_min_ = −0.16 e Å^−^^3^

### Special details

**Table:**

Geometry. All e.s.d.'s (except the e.s.d. in the dihedral angle between two l.s. planes) are estimated using the full covariance matrix. The cell e.s.d.'s are taken into account individually in the estimation of e.s.d.'s in distances, angles and torsion angles; correlations between e.s.d.'s in cell parameters are only used when they are defined by crystal symmetry. An approximate (isotropic) treatment of cell e.s.d.'s is used for estimating e.s.d.'s involving l.s. planes.
Refinement. Refinement of *F*^2^ against ALL reflections. The weighted *R*-factor *wR* and goodness of fit *S* are based on *F*^2^, conventional *R*-factors *R* are based on *F*, with *F* set to zero for negative *F*^2^. The threshold expression of *F*^2^ > σ(*F*^2^) is used only for calculating *R*-factors(gt) *etc*. and is not relevant to the choice of reflections for refinement. *R*-factors based on *F*^2^ are statistically about twice as large as those based on *F*, and *R*- factors based on ALL data will be even larger.

### Fractional atomic coordinates and isotropic or equivalent isotropic displacement parameters (Å^2^)

**Table:**

	*x*	*y*	*z*	*U*_iso_*/*U*_eq_	
O1	0.5813 (2)	0.56693 (10)	0.5900 (2)	0.0580 (6)	
O2	0.6448 (3)	0.60901 (12)	0.7932 (2)	0.0874 (9)	
O3	0.9341 (2)	0.53730 (12)	1.1294 (2)	0.0629 (7)	
O4	0.7329 (2)	0.53120 (12)	1.2114 (2)	0.0705 (7)	
N1	0.7776 (2)	0.49308 (12)	0.8877 (2)	0.0497 (7)	
H1A	0.8252	0.5093	0.9617	0.060*	
H1B	0.7247	0.5233	0.8425	0.060*	
H1C	0.8387	0.4780	0.8423	0.060*	
N2	0.5142 (2)	0.44461 (12)	0.6901 (2)	0.0515 (7)	
H2A	0.4887	0.4232	0.6164	0.062*	
H2B	0.5633	0.4790	0.6768	0.062*	
H2C	0.4370	0.4563	0.7182	0.062*	
N3	0.4687 (3)	0.68504 (14)	0.4928 (3)	0.0660 (8)	
N4	1.1006 (3)	0.56390 (14)	1.3686 (3)	0.0606 (8)	
C1	0.6809 (3)	0.43883 (15)	0.9164 (3)	0.0469 (8)	
H1	0.6048	0.4573	0.9593	0.056*	
C2	0.6076 (3)	0.40179 (15)	0.7922 (3)	0.0494 (8)	
H2	0.6844	0.3820	0.7519	0.059*	
C3	0.5124 (4)	0.34650 (16)	0.8301 (3)	0.0646 (10)	
H3A	0.4353	0.3652	0.8698	0.078*	
H3B	0.4664	0.3227	0.7503	0.078*	
C4	0.6042 (4)	0.29907 (18)	0.9290 (4)	0.0789 (11)	
H4A	0.5408	0.2657	0.9550	0.095*	
H4B	0.6754	0.2772	0.8865	0.095*	
C5	0.6839 (5)	0.33547 (18)	1.0523 (3)	0.0802 (12)	
H5A	0.6128	0.3529	1.1007	0.096*	
H5B	0.7474	0.3052	1.1108	0.096*	
C6	0.7750 (4)	0.39129 (16)	1.0134 (3)	0.0625 (10)	
H6A	0.8516	0.3733	0.9722	0.075*	
H6B	0.8219	0.4150	1.0931	0.075*	
C7	0.4020 (5)	0.74176 (19)	0.4510 (4)	0.0801 (12)	
H7	0.3641	0.7465	0.3600	0.096*	
C8	0.3859 (5)	0.79253 (18)	0.5318 (4)	0.0832 (12)	
H8	0.3372	0.8307	0.4977	0.100*	
C9	0.4430 (5)	0.78617 (18)	0.6647 (4)	0.0863 (13)	
H9	0.4344	0.8203	0.7231	0.104*	
C10	0.5136 (4)	0.72897 (17)	0.7119 (3)	0.0668 (10)	
H10	0.5546	0.7241	0.8024	0.080*	
C11	0.5224 (3)	0.67897 (15)	0.6232 (3)	0.0493 (8)	
C12	0.5894 (3)	0.61329 (16)	0.6723 (4)	0.0542 (9)	
C13	1.1815 (4)	0.58402 (19)	1.4852 (4)	0.0769 (11)	
H13	1.2833	0.5832	1.4958	0.092*	
C14	1.1234 (4)	0.60559 (18)	1.5892 (4)	0.0715 (11)	
H14	1.1846	0.6196	1.6678	0.086*	
C15	0.9757 (5)	0.60653 (18)	1.5775 (4)	0.0723 (11)	
H15	0.9338	0.6212	1.6474	0.087*	
C16	0.8879 (4)	0.58488 (17)	1.4573 (3)	0.0646 (10)	
H16	0.7861	0.5843	1.4463	0.078*	
C17	0.9540 (3)	0.56461 (14)	1.3564 (3)	0.0452 (8)	
C18	0.8658 (4)	0.54258 (15)	1.2225 (3)	0.0496 (8)	

### Atomic displacement parameters (Å^2^)

**Table:**

	*U*^11^	*U*^22^	*U*^33^	*U*^12^	*U*^13^	*U*^23^
O1	0.0663 (15)	0.0528 (14)	0.0526 (14)	0.0046 (11)	0.0071 (11)	−0.0075 (11)
O2	0.126 (2)	0.0742 (18)	0.0482 (16)	0.0264 (16)	−0.0142 (15)	−0.0071 (12)
O3	0.0525 (14)	0.0915 (18)	0.0459 (14)	−0.0065 (12)	0.0128 (11)	−0.0127 (12)
O4	0.0427 (14)	0.102 (2)	0.0660 (17)	−0.0010 (13)	0.0095 (12)	−0.0170 (13)
N1	0.0466 (16)	0.0592 (17)	0.0403 (15)	0.0014 (13)	0.0018 (12)	−0.0019 (12)
N2	0.0448 (15)	0.0642 (18)	0.0444 (16)	−0.0042 (13)	0.0061 (12)	−0.0097 (13)
N3	0.077 (2)	0.062 (2)	0.053 (2)	0.0113 (16)	−0.0005 (15)	−0.0010 (14)
N4	0.0504 (18)	0.078 (2)	0.0512 (18)	−0.0100 (15)	0.0052 (14)	−0.0030 (14)
C1	0.0436 (18)	0.055 (2)	0.0424 (19)	−0.0030 (16)	0.0109 (15)	0.0000 (15)
C2	0.0467 (18)	0.057 (2)	0.0450 (19)	0.0038 (16)	0.0096 (15)	−0.0014 (15)
C3	0.069 (2)	0.065 (2)	0.062 (2)	−0.014 (2)	0.0177 (19)	−0.0052 (18)
C4	0.105 (3)	0.061 (2)	0.072 (3)	−0.006 (2)	0.021 (2)	0.007 (2)
C5	0.111 (3)	0.069 (3)	0.061 (3)	0.002 (2)	0.018 (2)	0.013 (2)
C6	0.073 (2)	0.062 (2)	0.050 (2)	0.0062 (19)	0.0045 (17)	0.0067 (17)
C7	0.104 (3)	0.070 (3)	0.058 (3)	0.012 (2)	−0.002 (2)	0.001 (2)
C8	0.107 (3)	0.056 (3)	0.083 (3)	0.012 (2)	0.011 (3)	0.004 (2)
C9	0.132 (4)	0.048 (2)	0.081 (3)	0.005 (2)	0.025 (3)	−0.016 (2)
C10	0.086 (3)	0.054 (2)	0.058 (2)	−0.002 (2)	0.0076 (19)	−0.0057 (18)
C11	0.0482 (19)	0.050 (2)	0.050 (2)	−0.0064 (16)	0.0087 (16)	−0.0029 (16)
C12	0.052 (2)	0.057 (2)	0.052 (2)	−0.0021 (17)	0.0063 (17)	−0.0047 (18)
C13	0.059 (2)	0.106 (3)	0.062 (3)	−0.019 (2)	0.004 (2)	−0.004 (2)
C14	0.079 (3)	0.088 (3)	0.045 (2)	−0.022 (2)	0.006 (2)	−0.0053 (19)
C15	0.083 (3)	0.083 (3)	0.056 (3)	0.001 (2)	0.028 (2)	−0.0101 (19)
C16	0.054 (2)	0.087 (3)	0.056 (2)	−0.0048 (19)	0.0173 (19)	−0.0143 (19)
C17	0.046 (2)	0.0454 (19)	0.043 (2)	−0.0001 (15)	0.0074 (15)	0.0047 (14)
C18	0.044 (2)	0.053 (2)	0.051 (2)	0.0054 (16)	0.0081 (17)	−0.0018 (16)

### Geometric parameters (Å, °)

**Table:**

O1—C12	1.255 (3)	C4—H4A	0.9700
O2—C12	1.241 (3)	C4—H4B	0.9700
O3—C18	1.251 (3)	C5—C6	1.517 (5)
O4—C18	1.239 (3)	C5—H5A	0.9700
N1—C1	1.489 (3)	C5—H5B	0.9700
N1—H1A	0.8600	C6—H6A	0.9700
N1—H1B	0.8600	C6—H6B	0.9700
N1—H1C	0.8600	C7—C8	1.349 (5)
N2—C2	1.493 (3)	C7—H7	0.9300
N2—H2A	0.8600	C8—C9	1.359 (5)
N2—H2B	0.8600	C8—H8	0.9300
N2—H2C	0.8600	C9—C10	1.374 (5)
N3—C11	1.330 (4)	C9—H9	0.9300
N3—C7	1.337 (4)	C10—C11	1.376 (4)
N4—C13	1.337 (4)	C10—H10	0.9300
N4—C17	1.343 (4)	C11—C12	1.515 (4)
C1—C2	1.513 (4)	C13—C14	1.359 (5)
C1—C6	1.526 (4)	C13—H13	0.9300
C1—H1	0.9800	C14—C15	1.353 (5)
C2—C3	1.528 (4)	C14—H14	0.9300
C2—H2	0.9800	C15—C16	1.400 (4)
C3—C4	1.525 (4)	C15—H15	0.9300
C3—H3A	0.9700	C16—C17	1.368 (4)
C3—H3B	0.9700	C16—H16	0.9300
C4—C5	1.517 (5)	C17—C18	1.512 (4)
			
C1—N1—H1A	109.5	H5A—C5—H5B	108.1
C1—N1—H1B	109.5	C5—C6—C1	111.6 (3)
H1A—N1—H1B	109.5	C5—C6—H6A	109.3
C1—N1—H1C	109.5	C1—C6—H6A	109.3
H1A—N1—H1C	109.5	C5—C6—H6B	109.3
H1B—N1—H1C	109.5	C1—C6—H6B	109.3
C2—N2—H2A	109.5	H6A—C6—H6B	108.0
C2—N2—H2B	109.5	N3—C7—C8	124.5 (4)
H2A—N2—H2B	109.5	N3—C7—H7	117.8
C2—N2—H2C	109.5	C8—C7—H7	117.8
H2A—N2—H2C	109.5	C7—C8—C9	118.0 (4)
H2B—N2—H2C	109.5	C7—C8—H8	121.0
C11—N3—C7	117.1 (3)	C9—C8—H8	121.0
C13—N4—C17	117.1 (3)	C8—C9—C10	119.5 (3)
N1—C1—C2	112.9 (2)	C8—C9—H9	120.2
N1—C1—C6	108.0 (2)	C10—C9—H9	120.2
C2—C1—C6	109.3 (3)	C9—C10—C11	118.8 (3)
N1—C1—H1	108.9	C9—C10—H10	120.6
C2—C1—H1	108.9	C11—C10—H10	120.6
C6—C1—H1	108.9	N3—C11—C10	122.1 (3)
N2—C2—C1	113.2 (2)	N3—C11—C12	117.3 (3)
N2—C2—C3	108.8 (2)	C10—C11—C12	120.6 (3)
C1—C2—C3	109.8 (2)	O2—C12—O1	124.8 (3)
N2—C2—H2	108.3	O2—C12—C11	116.8 (3)
C1—C2—H2	108.3	O1—C12—C11	118.4 (3)
C3—C2—H2	108.3	N4—C13—C14	123.7 (4)
C4—C3—C2	111.0 (3)	N4—C13—H13	118.2
C4—C3—H3A	109.4	C14—C13—H13	118.2
C2—C3—H3A	109.4	C15—C14—C13	119.6 (3)
C4—C3—H3B	109.4	C15—C14—H14	120.2
C2—C3—H3B	109.4	C13—C14—H14	120.2
H3A—C3—H3B	108.0	C14—C15—C16	118.2 (3)
C5—C4—C3	110.7 (3)	C14—C15—H15	120.9
C5—C4—H4A	109.5	C16—C15—H15	120.9
C3—C4—H4A	109.5	C17—C16—C15	119.0 (3)
C5—C4—H4B	109.5	C17—C16—H16	120.5
C3—C4—H4B	109.5	C15—C16—H16	120.5
H4A—C4—H4B	108.1	N4—C17—C16	122.4 (3)
C6—C5—C4	110.6 (3)	N4—C17—C18	115.7 (3)
C6—C5—H5A	109.5	C16—C17—C18	121.9 (3)
C4—C5—H5A	109.5	O4—C18—O3	124.4 (3)
C6—C5—H5B	109.5	O4—C18—C17	119.0 (3)
C4—C5—H5B	109.5	O3—C18—C17	116.6 (3)
			
N1—C1—C2—N2	−59.0 (3)	C9—C10—C11—C12	−175.5 (3)
C6—C1—C2—N2	−179.2 (2)	N3—C11—C12—O2	177.0 (3)
N1—C1—C2—C3	179.2 (2)	C10—C11—C12—O2	−5.3 (5)
C6—C1—C2—C3	59.0 (3)	N3—C11—C12—O1	−5.1 (4)
N2—C2—C3—C4	177.0 (3)	C10—C11—C12—O1	172.6 (3)
C1—C2—C3—C4	−58.6 (4)	C17—N4—C13—C14	−0.9 (5)
C2—C3—C4—C5	56.3 (4)	N4—C13—C14—C15	0.9 (6)
C3—C4—C5—C6	−54.9 (4)	C13—C14—C15—C16	0.1 (6)
C4—C5—C6—C1	56.8 (4)	C14—C15—C16—C17	−0.9 (5)
N1—C1—C6—C5	177.9 (3)	C13—N4—C17—C16	0.1 (5)
C2—C1—C6—C5	−58.9 (4)	C13—N4—C17—C18	179.1 (3)
C11—N3—C7—C8	−0.1 (6)	C15—C16—C17—N4	0.8 (5)
N3—C7—C8—C9	1.0 (7)	C15—C16—C17—C18	−178.1 (3)
C7—C8—C9—C10	−0.4 (6)	N4—C17—C18—O4	167.7 (3)
C8—C9—C10—C11	−1.0 (6)	C16—C17—C18—O4	−13.2 (5)
C7—N3—C11—C10	−1.5 (5)	N4—C17—C18—O3	−12.5 (4)
C7—N3—C11—C12	176.2 (3)	C16—C17—C18—O3	166.5 (3)
C9—C10—C11—N3	2.0 (5)		

### Hydrogen-bond geometry (Å, °)

**Table:**

*D*—H···*A*	*D*—H	H···*A*	*D*···*A*	*D*—H···*A*
N1—H1A···O3	0.86	1.89	2.749 (3)	175
N1—H1B···O2	0.86	1.92	2.743 (3)	160
N1—H1C···O3^i^	0.86	2.10	2.790 (3)	137
N1—H1C···N4^i^	0.86	2.49	3.271 (4)	152
N2—H2A···O1^ii^	0.86	2.09	2.828 (3)	144
N2—H2A···N3^ii^	0.86	2.53	3.254 (4)	142
N2—H2B···O1	0.86	2.02	2.807 (3)	152
N2—H2C···O4^iii^	0.86	1.88	2.734 (3)	171

Symmetry codes: (i) −*x*+2, −*y*+1, −*z*+2; (ii) −*x*+1, −*y*+1, −*z*+1; (iii) −*x*+1, −*y*+1, −*z*+2.
